# Providence virus: An animal virus that replicates in plants or a plant virus that infects and replicates in animal cells?

**DOI:** 10.1371/journal.pone.0217494

**Published:** 2019-06-04

**Authors:** Meesbah Jiwaji, Gwynneth Felicity Matcher, Mart-Mari de Bruyn, Janet Awino Awando, Holisha Moodley, Dylan Waterworth, Rachel Anne Jarvie, Rosemary Ann Dorrington

**Affiliations:** Department of Biochemistry and Microbiology, Rhodes University, Grahamstown, South Africa; Wuhan Institute of Virology, Chinese Academy of Sciences, CHINA

## Abstract

**Introduction:**

Emerging viral diseases, most of which are zoonotic, pose a significant threat to global health. There is a critical need to identify potential new viral pathogens and the challenge is to identify the reservoirs from which these viruses might emerge. Deep sequencing of invertebrate transcriptomes has revealed a plethora of viruses, many of which represent novel lineages representing both plant and animal viruses and little is known about the potential threat that these viruses pose.

**Methods:**

Providence virus, an insect virus, was used to establish a productive infection in *Vigna unguiculata* (cowpea) plants. Providence virus particles purified from these cowpea plants were used to infect two mammalian cell lines.

**Findings:**

Here, we present evidence that Providence virus, a non-enveloped insect RNA virus, isolated from a lepidopteran midgut cell line can establish a productive infection in plants as well as in animal cells. The observation that Providence virus can readily infect both plants and mammalian cell culture lines demonstrates the ability of an insect RNA virus to establish productive infections across two kingdoms, in plants and invertebrate and vertebrate animal cell lines.

**Conclusions:**

The study highlights the potential of phytophagous insects as reservoirs for viral re-assortment and that plants should be considered as reservoirs for emerging viruses that may be potentially pathogenic to humans.

## Introduction

The threats posed by human viral diseases, many of which are caused by zoonotic viruses [1], are increasingly in the public eye. Recent deep sequencing approaches have highlighted the diversity of viruses, most of which are undescribed [2], leading to the possibility that unknown emerging viruses may pose a threat to human health. In this article, we highlight that phytophagous insects can act as reservoirs for viruses that could be pathogenic to humans.

Providence virus (PrV) is a small, non-enveloped insect RNA virus that was originally identified as a persistent infection in the corn earworm, *Helicoverpa zea* (*H*. *zea*), midgut cell line [3]. Like other tetraviruses, PrV was thought infect a narrow host range confined to closely related lepidopteran species including *Helicoverpa* and *Spodoptera* [3,4] but more recently, PrV has been shown to infect and persistently replicate in the human cervical cancer (HeLa) cell line [5]. It is interesting to note that deep sequencing has identified PrV-like transcripts in *Extatosoma tiaratum*, the giant prickly stick insect (NCBI nucleotide sequence GAWG01012535) and that cDNA-derived PrV transcripts are capable of launching PrV replication in insect, mammalian as well as plant cell-free extracts [5]. These observations raise questions about the host range of PrV.

**Fig 1:**
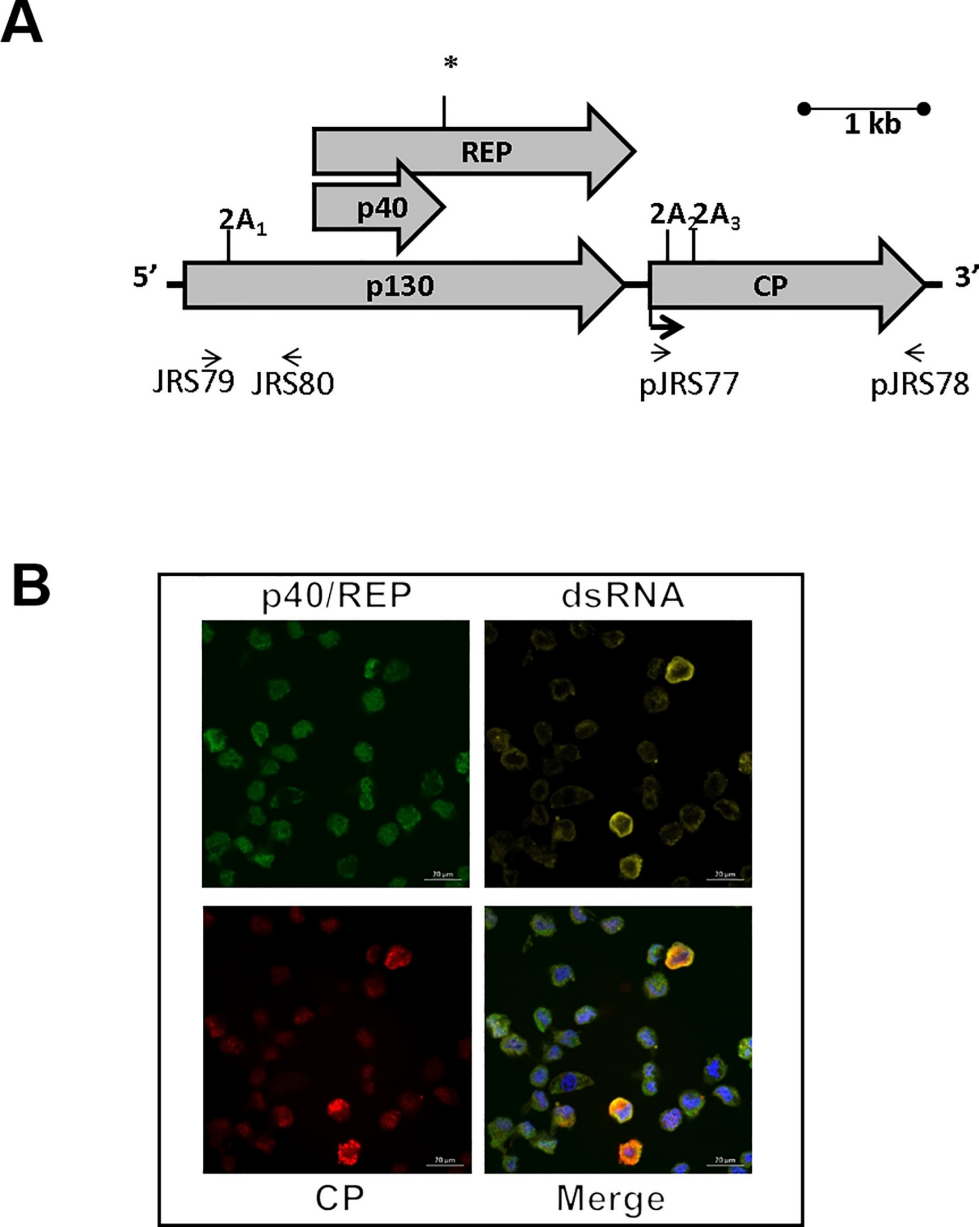
Providence virus in *Helicoverpa zea* MG8 cells. (a) Schematic diagram showing the genome organization of PrV. The three ORFs p130, REP (viral RdRp) and CP (viral capsid) are shown. The 2A-like processing sites (2A_1_, 2A_2_ and 2A_3_) are marked in p130 and CP. The (*) in REP indicates a readthrough stop codon resulting in the production of p40 (replication accessory protein) and REP from the same sequence. (b) Detection of p40/REP, dsRNA and the CP in insect *H*. *zea* MG8 cells. Cells were probed with mouse monoclonal anti-dsRNA and anti-mouse AF546 [8]. Viral replicase was stained with biotin-conjugated p40 and streptavidin AF488, while rabbit polyclonal anti-CP and anti-rabbit AF633 were used to detect PrV CP. All images represent 1 μm optical slices taken using a Zeiss LSM780 laser scanning confocal microscope using a X63, 0.75 NA objective. Scale bar represents 20 μm.

All tetraviruses have monopartite or bipartite (+) ssRNA genomes, encoding a viral replicase (REP) and a single, capsid protein precursor (CP), 240 copies of which assemble into a procapsid that is autoproteolytically cleaved during particle maturation [4]. In addition to REP and CP, PrV encodes a large ORF (p130) that overlaps with the REP ORF (Fig 1A). Unlike other tetraviruses, with alpha-like or picorna-like replicases, PrV has a carmo-like viral replicase that is most closely related to those of plant viruses belonging to the *Tombusviridae* family [4,6]. PrV and tombusviruses also share a similar translation strategy for the viral replicase, expressing a shorter accessory (p40) and full-length replicase protein (p104) from the same ORF via a read-through stop codon [6,7]. This suggests that PrV may have evolved via re-assortment between tombusvirus and tetravirus ancestors. The observation that cDNA-derived transcripts replicate *in vitro* in plant cell-free extracts [5] raised the possibility that PrV may have retained the ability to replicate in plants and in this study we set out to test this hypothesis.

## Materials and methods

### General culture conditions

MG8 cells were grown to confluence in complete ExCell 420 medium in T75 flasks. HeLa (ATCC CCL-2) and MCF7 (ATCC HTB-22) were maintained in DMEM supplemented with 10% FBS at 37°C in an atmosphere that contained 5% CO_2_.

### Virus particle purification

Cells were treated with 0.1% Triton X-100 for 15 mins. The supernatant was clarified at 5 000 rpm for 15 mins at 4°C. Virus particles were purified as described previously [9] by ultracentrifugation at 100 000xg for 3 hr through a 30% sucrose cushion and sucrose gradient density ultracentrifugation using a 10% - 40% sucrose gradient (w/v) at 140 000xg for 75 min. Virus was pelleted from selected gradient fractions by ultracentrifugation for 3.5 hr at 100 000xg. Pooled virus pellets were re-suspended overnight in 50 mM Tris buffer (pH 7.4) at 4°C and stored at -20°C in Tris buffer containing 20% glycerol (v/v).

### Infection of cowpea plants

Carborundum was sprinkled lightly over the two leaves of the cowpea plants. A gloved finger, wet in a buffer solution (mock infected plants) and PrV solution (infected plants) where PrV was diluted to approximately 10^5^ particles mL^-1^ was used to gently stroke the leaves. After 30 mins, the leaves were rinsed with water until no carborundum powder was visible on the leaves. The plants were grown in a 28°C constant environment room and watered as required. Watering regularly washed the leaves to ensure the removal of any PrV or carborundum on the leaf surface. After harvesting, plant material was stored at -80°C until analysis.

### Infection of mammalian cell lines

HeLa and MCF7 cells were grown to 50% confluence in a T25 flask. A total of 10^3^ particles were added to the cells and the flasks incubated until confluence. These cells were analysed by fluorescence microscopy as described below.

### cDNA and PCR

RNA was extracted with the RNA Shield Purification Kit (Zymo Research, R1100). A total of 10 ug RNA was used in reverse transcription reactions with the QuantiTect Reverse Transcription Kit (Qiagen, 205311) with random primers (supplied with the kit), with primers specific for the negative sense RNA (JRS79F- CGA GGT TAC CAC AAC CTG C or JRS77F- GTG GTA CCT CTA CAA ATG TGG TAT GGC TG) or the positive sense RNA (JRS80R- GAT GCC CTC GGC AAC C or JRS 78R- CTT GGC TCG TCT AGC GC). The cDNA was subsequently PCR-amplified for 30 cycles (with the primers JRS79-JRS80) or 53 cycles (with the primers JRS77-JRS78) using the KAPA Taq PCR Kit (Kapa, KK1006) as described previously [5]. The plasmid pFLM encoding the complete PrV genome (Genbank Accession Number KX280626.3) was used as a positive control).

### SDS-PAGE and Western blots

Protein extracts were separated on 10% SDS-PAGE gels and transferred to Immobilon-P membrane (Millipore) for western analysis with anti-CP anti-serum [6] (rabbit 1:10 000) and anti-rabbit HRP (goat 1:20 000; Advansta R-05072-500). Signal was detected with WesternBright ECL HRP substrate (Advansta K-12045-D50).

### Transmission electron microscopy

Sample preparation and negative staining was performed as previously described [10]. A total of 5 μL of a 0.1 mg mL^-1^ virion solution was allowed to adsorb to the grids for 2 min before staining [11]. Samples were observed with a Zeiss Libra 120.

### Fluorescence microscopy

Cells settled overnight on glass coverslips were washed three times in PBS (pH 7.0) for 5 min after which the cells were fixed in 4% paraformaldehyde for 15 min followed by three washes in PBS as above. The cells were permeabilized in permeabilization buffer (PBS containing 5% goat serum, 10% sucrose and 1% Triton X-100) for 15 min. The cells were incubated with anti-p40 [6] (biotin-linked antibody 1:1000), anti-dsRNA (clone K1 mouse 1:1000; Product Number 10020500 English & Scientific Consulting) and anti-CP anti-serum [6] (rabbit 1:1000) in permeabilization buffer for 90 min then incubated with 20 μg mL^-1^ anti-biotin AF488 (Invitrogen; S11223), anti-mouse AF546 (Invitrogen; A11003) and anti-rabbit AF633 (Invitrogen; A21070) in permeabilization buffer for 30 min. Cells were washed thrice, for 15 mins. In the second wash, 1 μg mL^-1^ DAPI was added to stain the nuclei of the cells. Cells were mounted using fluorescent mounting medium (DAKO, S3023). Cells were imaged using a Zeiss LSM780 laser scanning confocal microscope using the x63, 0.75 NA objective. All images were acquired using the same exposure and detector settings for each spectral channel. The Zen 2011 Blue software was used to acquire images from the Zeiss LSM780 microscope and to perform image overlays.

## Results

PrV particles were isolated from persistently-infected *H*. *zea* MG8 cells in which the virus had first been identified [3]. In these cells, virus replication factories can be visualized by immunofluorescence confocal microscopy in punctate, cytoplasmic structures that co-stain for double stranded vRNA (viral RNA) and the viral replicase (p40/REP). High concentrations of the CP, likely representing virus particles, are present in a sub-population (~10–25%) of cells (Fig 1B).

**Fig 2:**
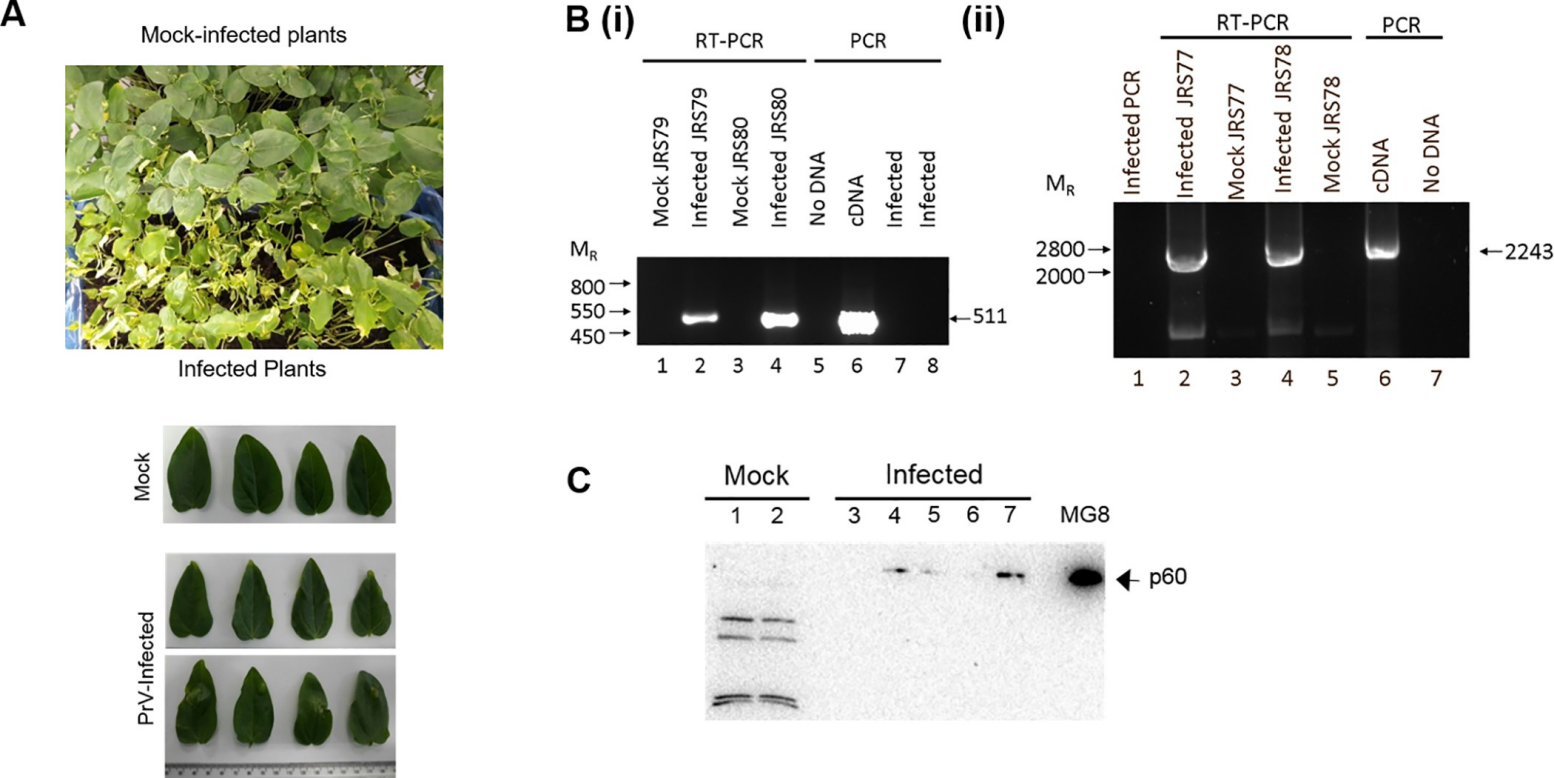
PrV replication in the cowpea plants. (a) Comparison of leaves from mock infected and PrV-infected cowpea plants. (b) RNA was isolated from macerated mock infected or PrV-infected leaves. Reverse transcription reactions were performed with specific primers to detect the (+)-sense (JRS80 or JRS78) or the (-)-sense (JRS79 or JRS77) RNA. The cDNA was probed for the presence of a 5’ region of the PrV genome or the CP sequence with the primers JRS79-JRS80 or JRS77-JRS78 respectively. For the control reactions, 1 μg pFLM (cDNA) was used. (c) Western analysis of the PrV proteins purified from individual leaves. Leaves from mock infected plants (lanes 1 and 2) and PrV-infected (lanes 3–7) were separated on an SDS-PAGE gel and western analysis performed with anti-PrV CP (rabbit) and anti-rabbit HRP (goat) antibodies. The sizes of the bands detected were compared to an extract from *H*. *zea* MG8 cells.

To determine whether PrV was infectious to plants, *Vigna unguiculata* (cowpea) seedlings, with two primary leaves, were mechanically infected with PrV particles purified from insect (MG8) cells. After two weeks, plants infected with PrV showed some lesions as well as yellowing of the leaves, which were not observed on the leaves of mock-infected plants (Fig 2A). This yellowing and stunting of PrV-infected plants became more noticeable after four weeks, when leaves were collected from the plants. Total RNA was purified from the mock infected and PrV-infected leaf extracts and vRNA was amplified by RT-PCR using strand-specific PCR primers that amplify regions at the 5’ terminal (JRS79 and JRS80) or the CP coding sequence (JRS77 and JRS78) of the PrV genome (Fig 1A). Amplification products corresponding to the 5’ region upstream of REP and the CP coding sequence were detected in the leaves of PrV-infected cowpea plants but not in the control plants (Fig 2B) The detection of both the (-) and (+) sense vRNAs confirmed that PrV is replicating in the plants.

**Fig 3:**
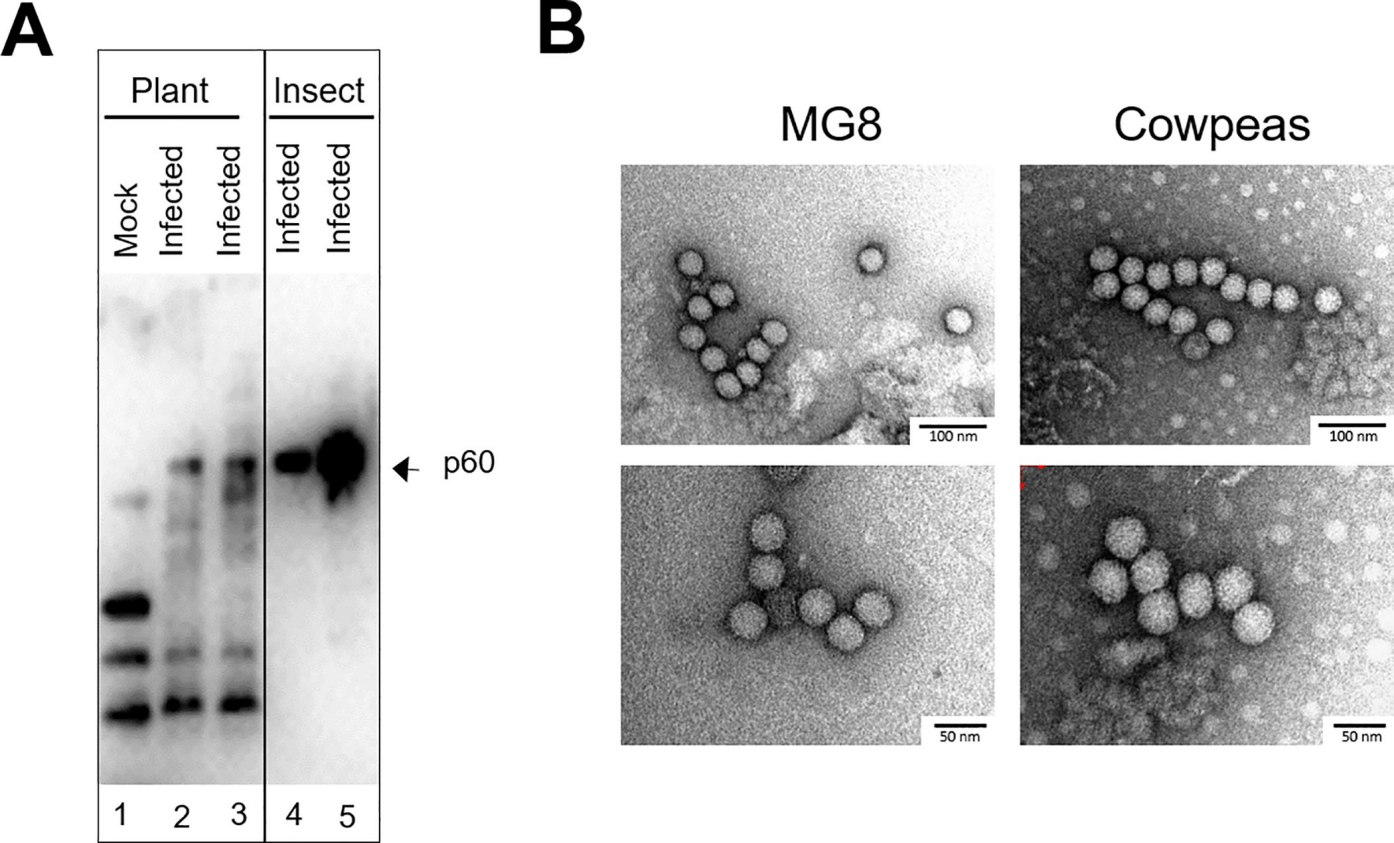
Detection of PrV in *Vigna unguiculata*, the cowpea plant. (a) Analysis of virus preparations from cowpea plants. Mock infected (lane 1) and PrV-infected cowpea plants (lanes 2 and 3) were separated on an SDS-PAGE gel and subjected to western analysis with anti-PrV CP (rabbit) and anti-rabbit HRP (goat) antibodies. The sizes of the bands detected were compared to the positive control, an extract from *H*. *zea* MG8 cells (lanes 4 and 5). (b) Visualization of the virus particles purified from *H*. *zea* MG8 cells and cowpea plants using transmission electron microscopy.

The observation of lesions and yellowing of leaves away from the site of infection suggested that the PrV infection might be able to spread throughout the plant. Consequently, individual leaves were collected away from the site of infection and analysed for the presence of PrV. Western analysis, using anti-CP antiserum, detected the presence of the major capsid protein (p60) in leaves collected from infected plants but not in those of mock-infected plants (Fig 2C) and all leaves from infected plants, irrespective of the location relative to the infection site, contained viral RNA (S1 Fig). This indicates that the PrV infection had moved from the original site of infection and had established itself throughout the cowpea plant. Virus particles were purified from PrV-infected plants by differential ultracentrifugation. Western analysis detecting the p60 capsid subunit (Fig 3A) and vRNA (S2 Fig) showed that both were present in the virus preparations from PrV infected plants, but not the mock infected plants. Finally, transmission electron microscopy revealed virus particles of approximately 40 nm in diameter and displaying a surface morphology consistent with that of PrV purified from the MG8 insect cell line (Fig 3B). These particles were absent in mock-infected cowpea plants.

**Fig 4:**
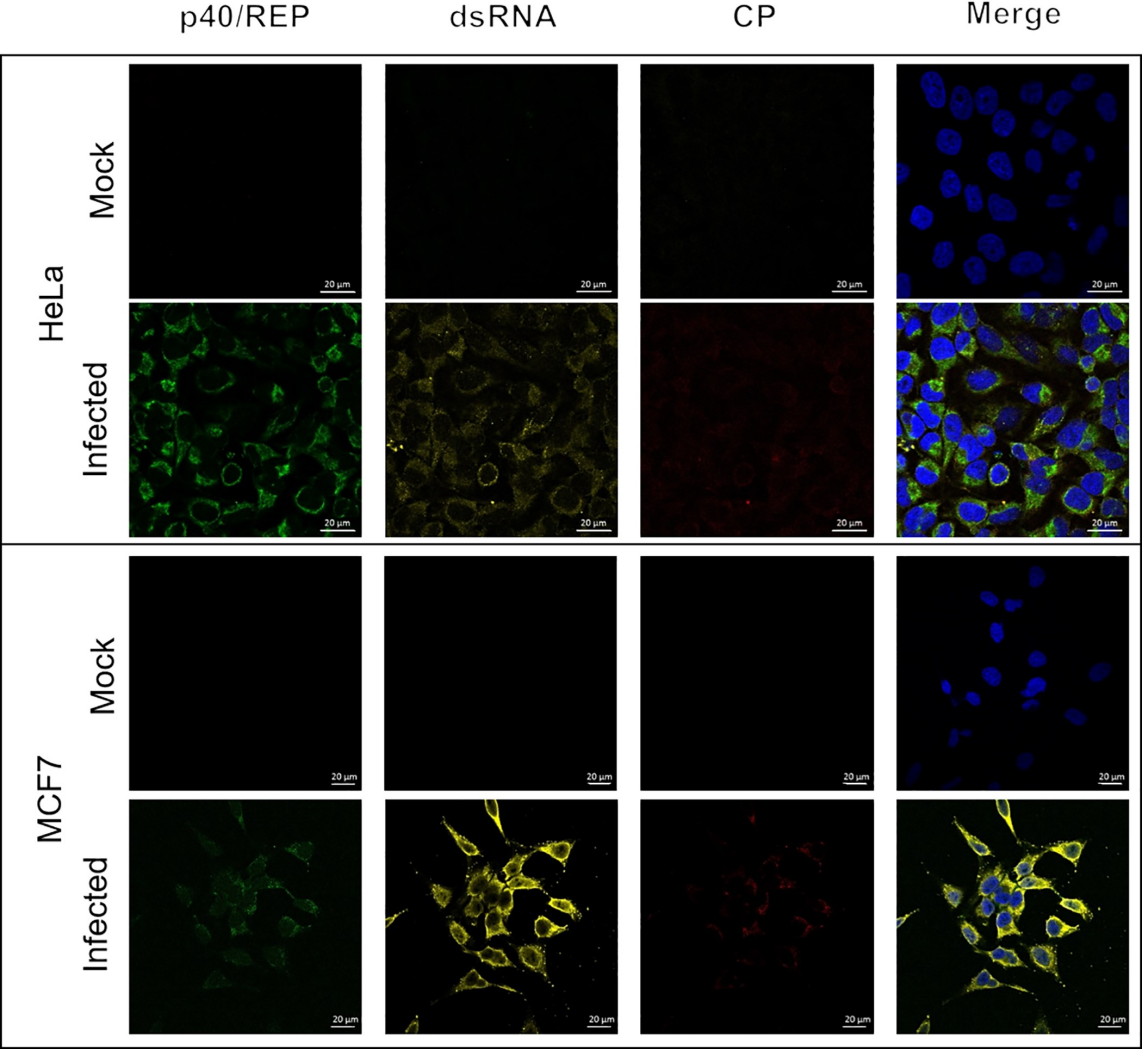
Infection of mammalian cells with PrV produced in cowpea plants. Human cervical (HeLa) and breast (MCF7) cancer cells were infected with PrV and subsequently analysed by immunofluorescence microscopy. Cells were probed with mouse monoclonal anti-dsRNA and anti-mouse AF546 [8]. Viral replicase was stained with biotin-conjugated p40 and streptavidin AF488, while rabbit polyclonal anti-CP and anti-rabbit AF633 were used to detect PrV CP. All images represent 1 μm optical slices taken using a Zeiss LSM 780 laser scanning confocal microscope using a X63, 0.75 NA objective. Scale bar represents 20 μm.

In order to be a true trans-kingdom virus, PrV must not only replicate in plants but must still retain its ability to infect and replicate in mammalian cells after being passaged through plant cells. To this end, virus particles prepared from cowpea plants were used to infect human cervical (HeLa) and breast (MCF7) cancer cells as described previously [5] and immunofluorescence microscopy was used to visualize viral replication. After 24 hours, the PrV REP, CP and dsRNA was detected in every cell for both cell lines (Fig 4), confirming that PrV purified from plants was infectious to the animal cells. After three months, replicating virus could still be detected in these cells, albeit at lower levels, indicating persistent PrV infections in these two mammalian cancer cell lines (S3 Fig).

## Discussion

In this study, we demonstrate the ability of an insect RNA virus to establish productive infections across two kingdoms, in plants as well as invertebrate and vertebrate animal model systems. Host shift, which is the ability of a virus to move from one host into a novel species, is often associated with changes in the sequence of the viral genome [12]. These changes can come at a cost to the pathogen’s fitness. It is therefore remarkable to us that PrV purified from plants can infect and establish persistent infections within mammalian cells. This implies that PrV produced in the *H*. *zea* MG8 cell line bind, enter, and successfully establish productive infections in mammalian cells while retaining the ability to replicate in plants. It would be interesting to analyse the genomes of PrV isolated from MG8 insect cells, cowpea plants as well as mammalian cell lines. There is growing evidence that the same changes are observed in the sequence of the viral genome when the virus infects a specific host [12]. PrV would provide an opportunity to perform studies on a virus that crosses kingdom boundaries and importantly, retains viral fitness.

In studies requiring the mechanical infection of plants with viruses, the conventional method involves the use of carborundum. In this study, we also used carborundum to inoculate cowpea plants with PrV particles. This method mimics the transfer of virus particles from biting insects to plant hosts and inversely this could represent the mechanism by which viruses are transferred from the plant to biting insects. Given the phylogenetic relationship between the PrV and tombusvirus replicases, it is probable that PrV evolved because of re-assortment between an ancestral tombus-like virus ingested by an *H*. *zea* larva infected with a tetra-like virus. This scenario is not unreasonable, given that the ability of heliothine midgut cells to absorb nucleic acids is well known [13] and is being exploited as a mechanism for applying RNAi technologies to control agricultural pests [14].

**Fig 5:**
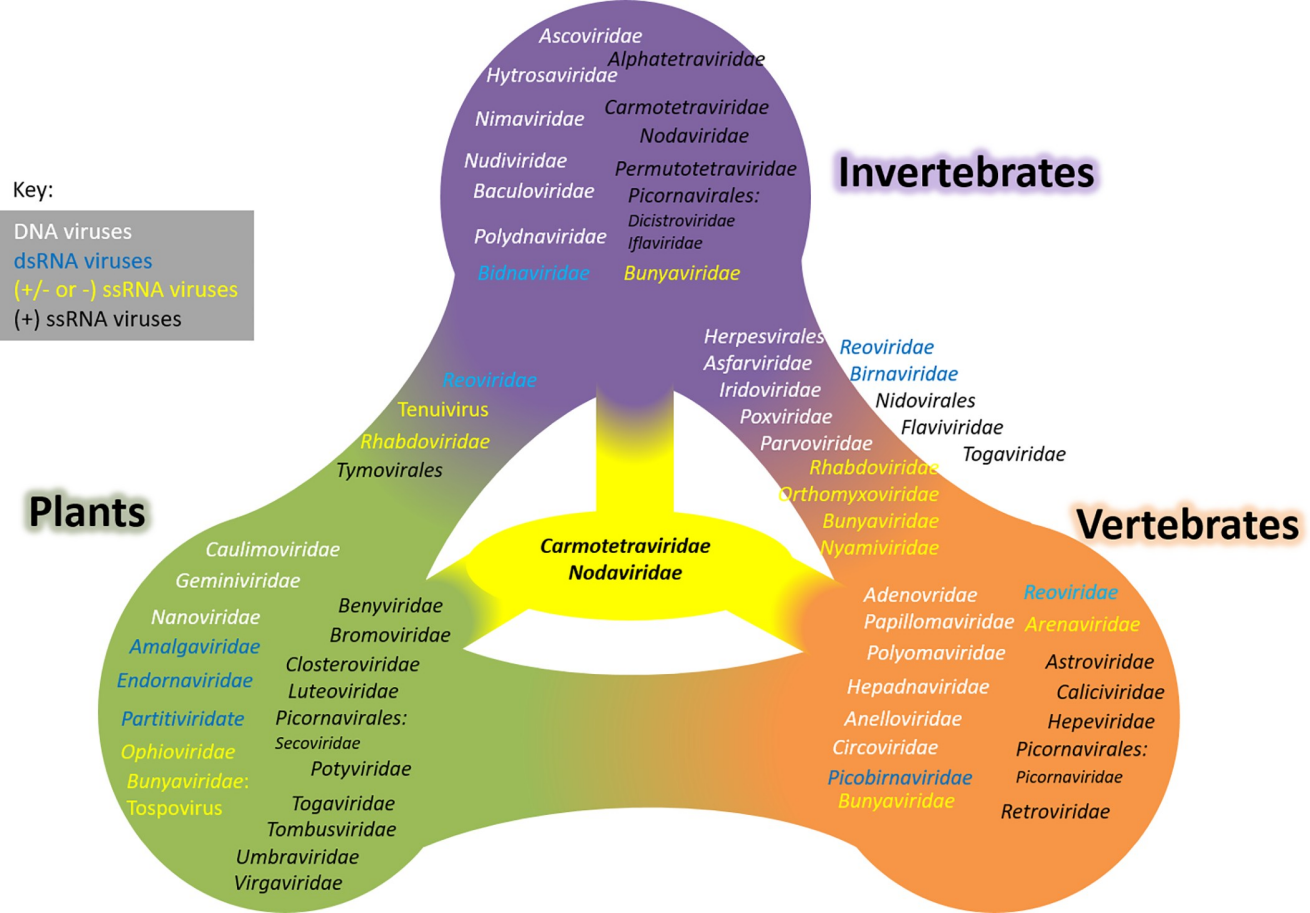
Diagrammatic representation of the host range of viruses. Invertebrate, plant and vertebrate-infecting virus families are shown in purple, green and orange respectively. The viruses infecting more than one type of host are positioned between the hosts. The *Nodaviridae* and the *Carmotetraviridae*, the only virus families that replicate in more than two types of host are shown in yellow in the centre of the figure. This figure is derived from the ICTV Master Species List version 1.1 updated April 6, 2017.

There are several families of viruses that infect invertebrates and vertebrates, including dsDNA viruses (*Asfarviridae*, *Iridoviridae*, *Poxviridae*), ssDNA viruses (*Parvoviridae*), dsRNA viruses belonging to the *Birnaviridae* family and numerous ssRNA viruses (the (-)-sense *Nyamiviridae*, *Orthomyxoviridae* and (+)-sense *Flaviviridae* and *Togaviridae*, (Fig 5). Viruses belonging to (+) ssRNA *Tymoviridae* and Tenuivirus are able to infect invertebrates and plants [15]. There are some virus families that have diverse host ranges. The *Reoviridae* (dsRNA) family includes viruses that infect vertebrates, vertebrates and invertebrates, or plants and invertebrates. The *Rhabdoviridae* (ssRNA) includes viruses that replicate in invertebrates and vertebrates or in invertebrates and plants. The (+/-)-ssRNA *Bunyaviridae* includes viruses that replicate in invertebrates, in vertebrates, in invertebrates and vertebrates, and in plants (Tospovirus). However, for all these virus families, no viruses have been reported to replicate in invertebrates, vertebrates as well as plants to date [15].

Members of the *Nodaviridae* are the only reported viruses which are known to infect both invertebrates and vertebrate hosts [15] and which can replicate successfully in cells derived from plants, insects and mammals (Fig 5). The alphanodavirus, Nodamura virus (NoV) established a productive infection when injected into the hemocoel of the wax moth *Galleria mellonella* or by subcutaneous injection into suckling mice [16]. NoV has also been shown to replicate in plant cells and in the yeast, *Saccharomyces cerevisiae* [17,18], but only via the introduction of viral RNA, by transfection or expression by recombinant plasmids. Furthermore, replication of NoV in BHK21 mammalian cells required the transfection of viral RNA [19]. The host range of NoV is as diverse as PrV however there are key differences. PrV particles are capable of infecting whole plants, moving away from the site of infection in the plant, and producing infectious particles in the plant host. In contrast, NoV could be isolated from virus-inoculated leaves but not at a distance from the point of infection [18]. PrV particles purified from infected cowpea plants retain infectivity, and can establish a productive infection in animal cell lines. NoV purified from infected plants has not been tested for infectivity towards mammalian cell lines or mammals. It is also interesting to note that attempts to establish a cell culture system for NoV replication were either not successful or were difficult to reproduce [20,21] whereas PrV readily establishes a persistent viral infection in insect and mammalian cell culture lines ([3,5],this study).

It is important to note that there is no information about the mechanism of infection with respect to insect and mammalian cells for NoV or PrV. This represents a significant void considering the broad host range of the members of the *Nodaviridae* and *Carmotetraviridae*. In their report, Bailey *et al*. [21] reported that there was no cytopathic effect in mammalian cells infected with NoV. In contrast, NoV is reported to be lethal when injected into mosquitoes and suckling mice [22,23]. It would be interesting to determine whether PrV can infect an animal, for example the invertebrate *H*. *zea* or the mammalian suckling mice. It would also be relevant to determine whether infection with PrV was localised to the site of infection or whether it could spread to the whole animal. These studies are beyond the scope of this research; however, the knowledge that PrV can infect and replicate in cell lines makes PrV a valuable model system to study viral host range and to develop potential therapeutic interventions to RNA virus infections.

Reports in the literature do exist that suggest the possibility that some tobamoviruses may be infectious to humans. For instance, detection of the pepper mild mottle virus (PMMoV) in human faecal samples was linked with clinical symptoms of viral infection, including fever and abdominal pain [24], but without demonstrating that PMMoV was the causative agent. Furthermore, tobacco mosaic virus (TMV) RNA has been isolated from the saliva of smokers but not from the saliva of non-smokers and has been cited as evidence that this would occur only if TMV was actively replicating in the cells of the smokers [25]. However, this remains to be experimentally confirmed. This study therefore provides the first experimental evidence for viruses originating from plants with the ability to infect and replicate in human cell lines. This raises the question of the possible emergence of other plant viruses that, like PrV, have crossed Kingdoms and have the potential to be human pathogens. Recently, metagenomics sequencing of the transciptomes of 220 invertebrate species has revealed the transcripts representing 1445 RNA viruses, many of which form new viral families [2]. This study highlights the potential of invertebrates as reservoirs of new and emerging viral diseases [26]. So, how could viruses such as PrV cross the invertebrate-vertebrate barrier? The genome of a highly conserved PrV isolate was recently reconstructed from the transcriptome of guano from a female western barbastelle bat (*Barbastella barbastellus*) in Hungary [27]. This bat species is insectivorous, feeding on heliothine larvae [28] and so it is likely that the bat acquired PrV by ingesting PrV-infected larval prey. Given that PrV is able to infect mammalian tissue culture cell lines, it is possible that that PrV may have been replicating in this bat; bats are known reservoirs of highly pathogenic zoonotic human pathogens. We propose that the focus on viral hosts detracts from the fact that viruses are capable of infecting and replicating in diverse hosts and care should be taken with the assumption that viruses that originate from a plant or insect do not represent a potential emerging human viral pathogen.

## Supporting information

S1 Fig Detection of PrV RNA in leaves of infected *Vigna unguiculata*. Detection of viral RNA in cDNA generated from mock infected (lanes 1 to 3) and PrV-infected (lanes 4 to 9) leaves from cowpea plants using the primers JRS79-JRS80. For the control reactions, virus preparations from mock infected plants and infected plants (lanes 10 and 11 respectively) were used. 1 μg pFLM (cDNA) was used as a positive control.
(TIF)

S2 Fig Detection of PrV RNA in virus particles purified from cowpea plants. RNA isolated from purified particles was converted to cDNA with random primers and then probed for the presence of a 5’ region of the PrV genome (i) or the CP sequence (ii) with the primers JRS79-JRS80 or JRS77-JRS78 respectively. For the control reaction, 1 μg pFLM was used.
(TIF)

S3 Fig Detection of a persistent PrV infection in mammalian cells. Human cervical (HeLa) and breast (MCF7) cancer cells that had been infected with PrV isolated from cowpea plants were cultured for three months and subsequently analysed by immunofluorescence microscopy. Cells were probed with mouse monoclonal anti-dsRNA and anti-mouse AF546 [8]. Viral replicase was stained with biotin-conjugated p40 and streptavidin AF488, while rabbit polyclonal anti-CP and anti-rabbit AF633 were used to detect PrV CP. All images represent 1 μm optical slices taken using a Zeiss LSM 780 laser scanning confocal microscope using a X63, 0.75 NA objective. Scale bar represents 20 μm.
(TIF)
