# Supplementary figures and images for: Providence virus: An animal virus that replicates in plants or a plant virus that infects and replicates in animal cells?
Source: PLoS One. 2019 Jun 4;14(6):e0217494. doi: 10.1371/journal.pone.0217494 (PMC6548363; doi:10.1371/journal.pone.0217494)

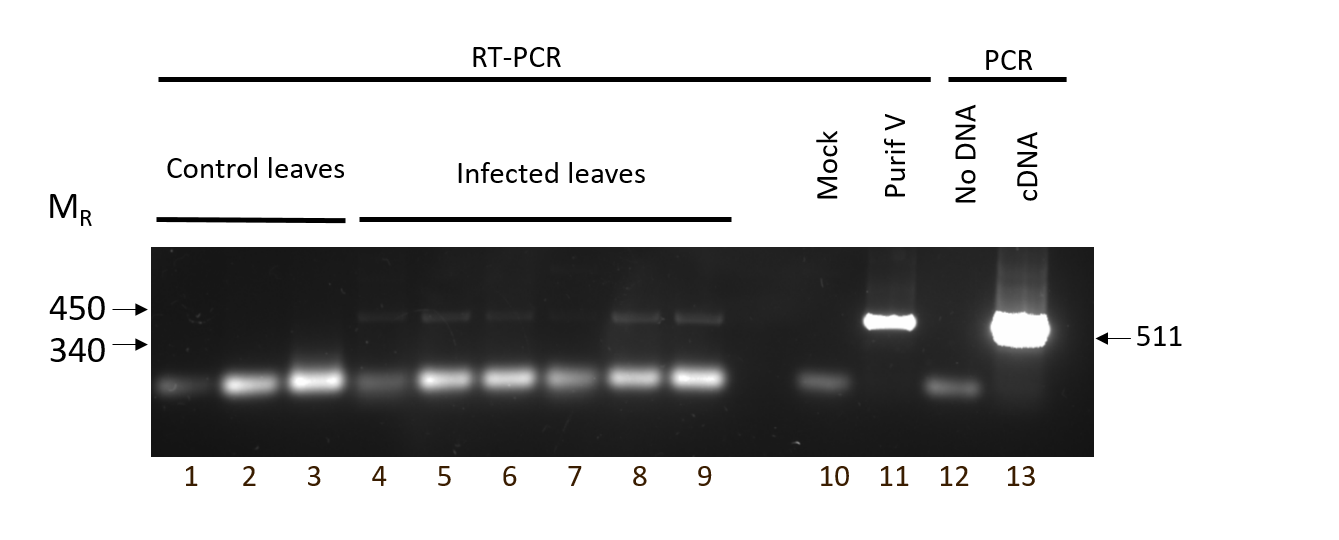

Supplement: S1 Fig — Detection of viral RNA in cDNA generated from mock infected (lanes 1 to 3) and PrV-infected (lanes 4 to 9) leaves from cowpea plants using the primers JRS79-JRS80. For the control reactions, virus preparations from mock infected plants and infected plants (lanes 10 and 11 respectively) were used. 1 μg pFLM (cDNA) was used as a positive control. (TIF) [file pone.0217494.s001.tif]

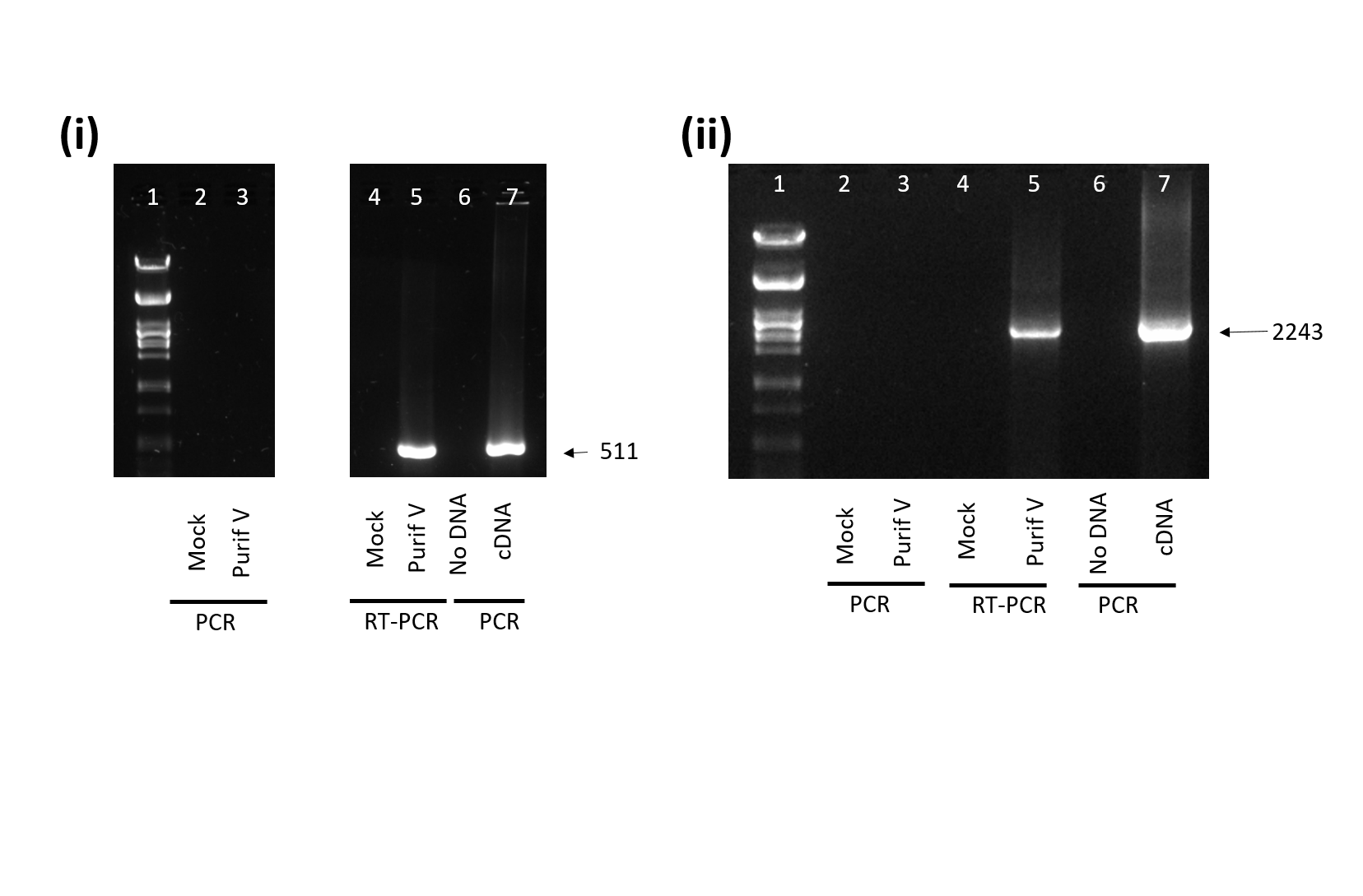

Supplement: S2 Fig — RNA isolated from purified particles was converted to cDNA with random primers and then probed for the presence of a 5’ region of the PrV genome (i) or the CP sequence (ii) with the primers JRS79-JRS80 or JRS77-JRS78 respectively. For the control reaction, 1 μg pFLM was used. (TIF) [file pone.0217494.s002.tif]

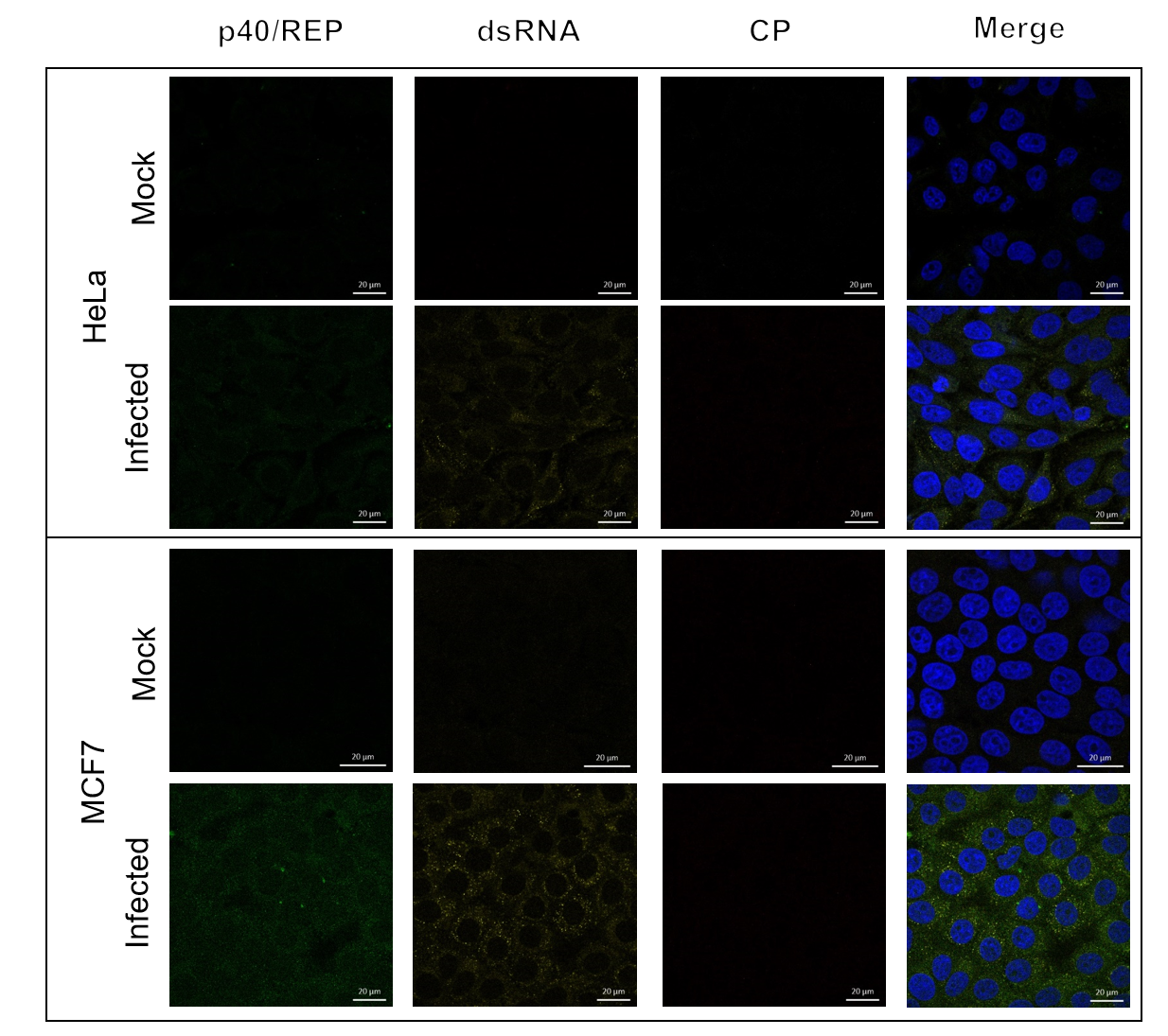

Supplement: S3 Fig — Human cervical (HeLa) and breast (MCF7) cancer cells that had been infected with PrV isolated from cowpea plants were cultured for three months and subsequently analysed by immunofluorescence microscopy. Cells were probed with mouse monoclonal anti-dsRNA and anti-mouse AF546 [8]. Viral replicase was stained with biotin-conjugated p40 and streptavidin AF488, while rabbit polyclonal anti-CP and anti-rabbit AF633 were used to detect PrV CP. All images represent 1 μm optical slices taken using a Zeiss LSM 780 laser scanning confocal microscope using a X63, 0.75 NA objective. Scale bar represents 20 μm. (TIF) [file pone.0217494.s003.tif]
